# Preliminary evidence of acylated ghrelin association with depression severity in postmenopausal women

**DOI:** 10.1038/s41598-021-84431-2

**Published:** 2021-03-05

**Authors:** Maria Fernanda Naufel, Amanda Paula Pedroso, Lila Missae Oyama, Mônica Marques Telles, Helena Hachul, Eliane Beraldi Ribeiro

**Affiliations:** 1 Department of Physiology, Universidade Federal de São Paulo (UNIFESP), Rua Botucatu 862, Vila Clementino, São Paulo, SP 04023-062 Brazil; 2 Department of Psychobiology, Universidade Federal de São Paulo (UNIFESP), São Paulo, SP Brazil; 3 Department Gynecology, Universidade Federal de São Paulo (UNIFESP), São Paulo, SP Brazil

**Keywords:** Gastrointestinal hormones, Biomarkers, Gastroenterology, Health care, Medical research, Neurology, Risk factors, Signs and symptoms, Diseases, Neurological disorders, Nutrition disorders, Psychiatric disorders, Neuroscience, Blood-brain barrier, Emotion, Physiology, Ageing, Neurophysiology

## Abstract

We have previously shown increased depression and anxiety scores in postmenopausal overweight women, when compared to overweight premenopausal women. The mechanisms responsible for these alterations are not understood. Although ghrelin involvement in mood modulation has been suggested, its role is still ambiguous and has not been evaluated in postmenopause. Here we investigated the association of ghrelin with depression and anxiety symptoms in postmenopausal women. Fifty-five postmenopausal women with depression symptoms, who were not in use of hormones or antidepressants, were included in the study. Depression symptoms were evaluated by Beck’s Depression Inventory (BDI) and Patient Health Questionnaire-9 (PHQ-9) and anxiety symptoms were evaluated by Beck’s Anxiety Inventory (BAI). Women were allocated into three groups, according to BDI classification of mild, moderate, or severe depression symptoms. Anthropometric, biochemical and hormonal parameters were analyzed. Total and acylated ghrelin levels were higher in the severe depression than in the mild depression group. Multivariate regression analyses showed positive associations of BDI scores with acylated ghrelin and BMI, and of PHQ-9 scores with acylated ghrelin and homeostasis model assessment of insulin resistance (HOMA-IR). BAI scores associated positively with waist-to-hip ratio. To the best of our knowledge, this is the first demonstration of an association between acylated ghrelin and the severity of depression symptoms in postmenopausal women. This association may reflect either a physiological response aimed at fighting against depression symptoms or a causal factor of this mental disorder.

## Introduction

The postmenopause-associated symptoms impact negatively women’s quality of life. We have previously found clinical and experimental evidence of a connection between the lack of ovarian hormones and increased rates of obesity, anxiety and depression^1–3^. Additionally, a bidirectional association between obesity and depression has been suggested^4,5^. The factors involved in the association of these disturbances in postmenopausal women have not been fully explored.

Ghrelin is a gastrointestinal hormone secreted mainly by the gastric mucosa cells that signals peripheral energy status to the brain, having an orexigenic effect dependent on the activation of the type 1a growth hormone secretagogue receptor (GHSR1a). Ghrelin circulates in two major forms, namely des-acyl and acyl ghrelin, the latter having a medium chain fatty acid attached to its serine 3 residue, a posttranslational modification essential for its binding to GHSR1a^6,7^. Although des-acyl ghrelin has been first considered an inactive molecule, it has later been suggested that both forms are functional, although with distinct roles^8^. Besides its role in growth hormone release and regulation of energy homeostasis, ghrelin has been shown to participate in a myriad of physiological processes including glucose homeostasis, neurogenesis, sleep/awake rhythm, learning and memory, stress responses, among others^9^.

Stimulation of food intake and reduction of energy expenditure and lipid oxidation have been described as effects of acylated ghrelin^10–12^ and the involvement of hypothalamic neurons in these actions has been demonstrated^13,14^. Interestingly, it has been shown that des-acyl ghrelin impairs the orexigenic effects of acyl ghrelin at the hypothalamus^15,16^. Ghrelin also takes part in the hedonic regulation of food intake, through actions in several brain regions implicated in the food reward-associated behavior^17–19^.

Ghrelin involvement in modulation of mood disorders has also been investigated but its role in depression and anxiety is still ambiguous. Although some studies in rodents have shown antidepressant and anxiolytic effects of total and acylated ghrelin^20–22^, other reports have evidenced depressogenic and anxiogenic roles^23–25^. Clinical data about ghrelin levels in depressive patients are also controversial. While some studies have reported no changes in acylated ghrelin levels in depressive patients^26^, others have reported decreased^27^ or increased levels^28^. The clinical heterogeneity observed in major depressive disorder might contribute, at least in part, to these inconclusive results. An association between total ghrelin levels and eating disorders in depressive individuals has been shown^29^. Intriguingly, among depressive patients, the ones who were experiencing increased appetite presented lower acylated ghrelin levels than both the ones reporting decreased appetite and the healthy subjects^30^.

Reduced serum total and acylated ghrelin has been observed in postmenopausal women^31–33^ and estrogen replacement therapy was able to increase acylated ghrelin levels^34^ and to improve hypothalamo-pituitary sensitivity to acylated ghrelin^35^. Weight loss has also been shown to raise total ghrelin levels in this population^36^. In rodents, the involvement of estrogens in the regulation of total ghrelin expression has been indicated^37^ and an antidepressant-like effect induced by ghrelin in ovariectomized mice has been shown^38^.

We were not able to find any studies investigating ghrelin’s association with mood disorders in postmenopausal women. A better comprehension of the postmenopause-associated changes and the factors influencing them will certainly improve the management of the symptoms affecting the quality of life of middle-aged women. In the present study, we hypothesized that ghrelin influences the depression and anxiety symptoms in postmenopausal women.

## Results

Postmenopausal women that were classified with mild depression according to BDI showed lower scores for anxiety symptoms (BAI scores, H_(2, n=55)_ = 21.20006, p < 0.0001) than the groups classified with moderate (p = 10^–6^) or severe depression (p < 10^–7^) (Table 1).

**Table 1:** Depression (BDI and PHQ-9) and anxiety (BAI) scores.

BDI classification	Mild depression *n* = 26	Moderate depression *n* = 22	Severe depression *n* = 7	p-value
BDI	12 (10–16)	21 (17–29)*	36 (32–37)*	**< 0.0001**
BAI	10 (3–25)	21.5 (7–43)*	31 (15–43)*	**< 0.0001**
PHQ-9	7.8 ± 0.8	15.8 ± 1.2*	22.9 ± 1.4*	**< 0.0001**

Variables with normal distribution were analyzed by ANOVA and presented as mean ± standard error. Variables not normally distributed were analyzed by Kruskall–Wallis test and presented as median (minimum–maximum).

*Vs. mild depression.

Bold values indicate statistical significance of *p* < 0.05.

There were no significant differences in anthropometric parameters among the 3 groups (Table 2). As shown in Table 3, the severe depression group had higher levels of total and acylated ghrelin than those of the mild depression group (H_(2, n=50)_ = 6.17622, p = 0.045 and H_(2, n=49)_ = 8.63559, p = 0.013, respectively). All other clinical parameters failed to show significant differences among the 3 groups.

**Table 2:** Anthropometric characteristics.

BDI classification	Mild depression *n* = 26	Moderate depression *n* = 22	Severe depression *n* = 7	p-value
Age (years)	57.3 ± 0.9	57.6 ± 0.8	55.4 ± 1.2	0.486
Age of menopause onset (years)	49.0 (40–56)	50.0 (38–55)	47.5 (37–52)	0.916
Years in menopause	7.8 (1–24)	8.0 (2–25)	7.9 (3–20)	0.585
BMI (kg/m^2^)	27.6 (18.9–34.2)	29.8 (22.7–38.5)	29.6 (21.2–38.1)	0.085
Body fat (kg)	25.4 (11–55)	32.1 (19–70)	31.5 (15–45)	0.081
Percentage body fat	38.5 ± 1.4	41.8 ± 1.4	41.9 ± 3.0	0.244
Skeletal muscle mass (Kg)	22.3 ± 0.7	25.1 ± 0.9	23.2 ± 1.8	0.101
Fat free mass (Kg)	41.5 ± 1.3	44.8 ± 1.3	41.6 ± 2.3	0.142
Waist/hip ratio	0.94 ± 0.01	0.97 ± 0.01	0.99 ± 0.02	0.093
Basal metabolic rate (Kcal)	1261.6 ± 27.2	1354 ± 28.06	1289 ± 62.0	0.132

Variables with normal distribution were analyzed by ANOVA and presented as mean ± standard error. Variables not normally distributed were analyzed by Kruskall–Wallis test and presented as median (minimum–maximum).

**Table 3:** Clinical data.

BDI classification	Mild depression	Moderate depression	Severe depression	p-value
Total cholesterol (mg/dL)	204.1 ± 8.4 [26]	210.9 ± 9.2 [21]	200.0 ± 16.5 [7]	0.792
HDL (mg/dL)	59.3 ± 3.3 [20]	62.1 ± 4.6 [14]	55.2 ± 8.0 [5]	0.696
LDL (mg/dL)	120.5 (79–187) [20]	131.0 (59–171) [14]	114.5 (97–140) [4]	0.829
Non-LDL (mg/dL)	149.1 ± 8.4 [20]	148.1 ± 13.1 [14]	135.5 ± 7.1 [4]	0.821
Triglycerides (mg/dL)	89.5 (37–261) [24]	114.5 (60–191) [18]	128.0 (69–193) [7]	0.167
Glucose (mg/dL)	91.0 (76–155) [25]	97.0 (83–149) [20]	98.0 (96–180) [6]	0.112
Insulin (μU/mL)	7.0 ± 0.7 [24]	8.9 ± 0.9 [22]	7.5 ± 1.5 [7]	0.259
HOMA-IR	1.66 (0.37–3.81) [23]	1.86 (0.95–5.29) [20]	2.01 (0.72–4.91) [6]	0.256
HOMA-ß	72.3 (30.8–163.8) [23]	95.7 (36.1–146.2) [20]	52.2 (26.5–138.8) [6]	0.277
Cortisol (ng/mL)	5.5 (2.4–12) [24]	4.8 (1.6–24.3) [20]	4.3 (3.7–6.1) [5]	0.511
FSH (mUI/mL)	85.5 (38–149) [26	78.1 (33–120) [22]	78.9 (41–118) [7]	0.838
Leptin (ng/mL)	34.3 (3.4–98.6) [26]	35.9 (7.9–60.6) [20]	26.9 (3.7–56.4) [7]	0.686
Total ghrelin (pg/mL)	355.8 (149–758) [24]	445.1 (283–997) [20]	557.1 (409–1406)* [6]	**0.045**
Acylated ghrelin (pg/mL)	85.7 (23–391) [24]	91.7 (17–445) [18]	222.4 (75–907)* [7]	**0.013**
Adiponectin (ng/mL)	2.94 (1.12–7.2) [26]	2.75 (1.0–5.5) [21]	3.40 (1.4–3.6) [7]	0.566

Variables with normal distribution were analyzed by ANOVA and presented as mean ± standard error [n]. Variables not normally distributed were analyzed by Kruskall–Wallis test and presented as median (minimum–maximum) [n].

*Vs. mild depression.

Bold values indicate statistical significance of *p* < 0.05.

The Pearson’s correlations were calculated including all women and all the variables assessed in the study, which included psychological scores, metabolic hormones and anthropometric measurements. Table 4 shows only the variables having at least one significant correlation with the psychological scores. A complete table containing all the correlations across all parameters is included as supplementary material (Supplementary Table 1).

**Table 4:** Pearson correlation coefficients

	Correlations					
	BDI		BAI		PHQ-9	
	Pearson correlation	p-value (2-tailed)	Pearson correlation	p-value (2-tailed)	Pearson correlation	p-value (2-tailed)
Total ghrelin	0.4841	**< 0.001**	0.2377	0.096	0.3380	**0.016**
Acylated ghrelin	0.5529	**< 0.001**	0.1373	0.347	0.3400	**0.017**
Body mass index	0.2558	**0.059**	0.2180	0.110	0.2280	0.093
Skeletal muscle mass	0.1358	0.303	0.2909	**0.031**	0.1359	0.322
Waist/hip ratio	0.3474	**0.009**	0.4442	**0.001**	0.4142	**0.002**
Fat free mass	0.1364	0.330	0.2970	**0.031**	0.1376	0.326
Basal metabolic rate	0.1533	0.264	0.2905	**0.031**	0.1327	0.334
Insulin	0.0579	0.680	0.2460	0.076	0.3715	**0.006**
Glucose	0.2301	0.104	0.2109	0.137	0.2769	**0.049**
HOMA-IR	0.1629	0.264	0.2686	0.062	0.4315	**0.002**

Only the variables showing at least one significant correlation are shown.

Bold values indicate statistical significance of *p* < 0.05.

BDI scores correlated positively with both total and acylated ghrelin levels and with WHR, and presented a tendency to a positive correlation with body mass index (BMI). BAI scores correlated positively skeletal muscle mass (SMM), waist-hip ratio (WHR), fat free mass (FFM), and basal metabolic rate (BMR). PHQ-9 scores correlated positively with total and acylated ghrelin and with WHR, insulin, glucose, and homeostasis model assessment of insulin resistance (HOMA-IR) (Table 4).

The linear regression models for BDI, BAI and PHQ-9 are shown in Tables 5, 6 and 7, respectively. For BDI as the dependent variable (Table 5), the independent variables acylated ghrelin, BMI, and WHR were tested. Total ghrelin was not included due to its multicollinearity with acylated ghrelin. We found that WHR failed to show a significant influence. The final model explained 38% of BDI variations (2 predictors, statistical power = 0.99, effect size = 0.64) and showed that acylated ghrelin levels and BMI were positively associated with depression symptoms (BDI scores). The final model implies that, for every one-unit increase in BMI, the BDI score increases 0.52 (95% CI 0.08–0.96), and that for every one-unit increase in acylated ghrelin the BDI score increases 0.027 (95% CI 0.018–0.042).

**Table 5:** Linear regression model for predictors of BDI among 49 postmenopausal women.

Dependent variable: BDI			
Predictors	Beta coefficients	Standard error	p-value
Acylated ghrelin	0.027	0.0055	**< 0.001**
Body mass index	0.526	0.2164	**0.019**
*Intercept*	− 0.179	6.3668	0.977

R = 0.62, R^2^ = 0.38, adjusted R^2^ = 0.36, F_(2,46)_ = 14.38, p < 0.0001.

The model was adjusted for waist/hip ratio (p = 0.395).

Bold values indicate statistical significance of *p* < 0.05.

**Table 6:** Linear regression model for predictors of BAI among 53 postmenopausal women.

Dependent variable: BAI			
Predictors	Beta coefficients	Standard error	p-value
Waist/hip ratio	81.07	22.89	**0.0008**
*Intercept*	− 59.54	21.93	**0.0090**

R = 0.44, R^2^ = 0.20, adjusted R^2^ = 0.18, F_(1,51)_ = 12.54, p < 0.01.

The model was adjusted for skeletal muscle mass (p = 0.678), basal metabolic rate (p = 0.577), and insulin (p = 0.354).

Bold values indicate statistical significance of *p* < 0.05.

**Table 7:** Linear regression model for predictors of PHQ-9 among 44 postmenopausal women.

Dependent variable: PHQ-9			
Predictors	Beta coefficients	Standard error	p-value
Acylated ghrelin	0.015	0.006	**0.008**
HOMA-IR	2.369	0.806	**0.005**
*Intercept*	5.770	2.070	0.008

R = 0.51, R^2^ = 0.26, adjusted R^2^ = 0.23, F_(2,41)_ = 7.31, p < 0.002.

The model was adjusted for waist/hip ratio (p = 0.109).

Bold values indicate statistical significance of *p* < 0.05.

For BAI scores, SMM, BMR, and insulin showed non-significant influence. Due to multicollinearity, we tested SMM and not FFM. The final model equation predicted 18% of BAI scores variations (1 predictor, statistical power = 0.99, effect size = 0.19) and showed that only WHR was positively associated with BAI scores (Table 6). The model implies that every 0.1 unit of WHR increase (95% CI 27.8–119.6) is associated with 8.1 increases in BAI scores.

For PHQ-9 as the dependent variable, WHR showed non-significant influence. Due to multicollinearity between total and acylated ghrelin and among glucose, insulin, and HOMA-IR variables, we selected acylated ghrelin and HOMA-IR for the regression analyses. The final model explained 26% of PHQ-9 variations (2 predictors, statistical power = 0.94, effect size = 0.35), showing positive association of acylated ghrelin and HOMA-IR with PHQ-9 scores (Table 7). The final model implies that for every one-unit increase in HOMA-IR the PHQ-9 score increases 2.37 (95% CI 0.73–4.01), and that for every one-unit increase in acylated ghrelin the PHQ-9 score increases 0.015 (95% CI 0.008–0.032).

## Discussion

The present results indicated that, after menopause, depressive and anxious symptoms are associated with both acylated ghrelin levels and parameters indicative of obesity and insulin resistance.

Among the postmenopausal women evaluated in the present study, all having some degree of depressive symptoms, we observed that the anxiety symptoms were higher in the groups with moderate and severe depression, when compared to the mild depression group. Moreover, the BAI scores correlated positively with those of the BDI and PHQ-9 inventories. These data are in agreement with previous studies reporting a bidirectional association between depression and anxiety symptoms in the general population^39–41^. Suggested mechanisms for this association emphasized the existence of common neurobiological basis for anxiety and depression symptoms, including abnormal activity of the hypothalamic–pituitary–adrenal axis (HPA axis), dysfunction of the γ-amino butyric acid (GABA) system, increased oxidative stress, and disturbance of neural plasticity^42–44^.

The three groups of depressive postmenopausal women presented median values of body parameters categorized as overweight, abdominal obesity, and high percentage of body fat^45^ (WHO, 2008), in agreement with previous reports^46,47^. These alterations have been associated with metabolic, sleep, cardiovascular, and psychological disorders in postmenopausal women^1,46,48^. These considerations emphasize the importance of monitoring the anthropometric parameters and encouraging healthy lifestyle habits in order to avoid weight gain in this population.

Moreover, an important influence of total and abdominal obesity on mental disorders has been described in middle-aged women^1^ as well as in the general adult population^49^. Here, we demonstrate such a connection in postmenopausal women, as WHR associated positively with BAI scores and BMI associated positively with BDI scores. Also, PHQ-9 scores associated with HOMA-IR. These findings indicate that obesity has a relevant influence on psychological symptoms in postmenopausal women.

In the general population, the deleterious influence of obesity exerts on psychological status has been associated with production of inflammatory mediators, stimulation of the HPA axis, and insulin resistance. Additionally, a role has been attributed to the dissatisfaction with body image and low self-esteem^49–51^. These mechanisms are likely involved in the pathogenesis of anxiety and depression in postmenopause with overweight. Additionally, impairment of monoaminergic function due to loss of estrogens could be relevant^52,53^. Stressor agents typical of this period of women`s life may also play a part, such as changes in social roles, financial insecurity, and empty nest syndrome^48,54,55^.

Increased incidence of depression has also been demonstrated in type I and type II adult diabetic patients^56^. A relevance to this association has been attributed to the central actions of insulin, since the hormone mediates neuromodulatory, neuroprotective and neurotrophic effects in the brain^57^. Impairment of central insulin action has been associated with cognitive impairment and depression, through mechanisms involving alterations in mitochondrial function, monoamine oxidase expression, and dopamine turnover^58^.

In the present study, we found that total and acylated ghrelin levels were significantly higher in the group with severe depression, in comparison to the group with mild depression, and that both total and acylated ghrelin levels correlated positively with both BDI and PHQ-9 scores. Moreover, in the linear regression analyses, acylated ghrelin appeared as an independent factor associated with depression symptoms in both questionnaires. This is the first study showing that the higher the serum acylated ghrelin levels, the more severe the depression symptoms in depressed postmenopausal women.

Although an association of depression symptoms and ghrelin levels has been studied in distinct populations, whether there is a positive or a negative relationship between them has not been established. In eutrophic young adults, elevated levels of acylated and des-acyl ghrelin were described in patients with severe and moderate depression^28^ and in individuals evaluated after suicide attempts^59^. In both eutrophic and overweight depressive adult patients, total ghrelin levels were higher than those in the respective non-depressive controls and showed an important reduction after depression treatment^60,61^. Reduction of the hormone levels after citalopram has also been reported in young eutrophic adults who, however, had pre-treatment levels of both acylated and des-acylated ghrelin even lower than those of the control group^27^. In contrast, there are reports of normal total and acylated ghrelin levels in overweight adults diagnosed with depression^62^ or eutrophic adults showing depressive symptoms^26,63^. Studies focusing on the ghrelin/depression association in women only are scarce. In eutrophic adults, total ghrelin levels were positively correlated with depression incidence in female but not in male individuals^64^.

The presence of a positive association of ghrelin and depression, as evidenced in the present study, could reflect either a physiological response aimed at fighting against depression symptoms or a causal factor of this mental disorder. Unfortunately, the available data are not conclusive. In healthy male rats, either juvenile or adult, the intracerebroventricular administration of total and acylated ghrelin increased depressive-like behaviors^23,25^, implicating the hormone as a possible pathophysiological mechanism for depression. A study in humans reported that the administration of acylated ghrelin to 7 eutrophic adults with unmedicated major depression failed to significantly modify depressive symptoms in both men and women^65^. At the best of our knowledge, no other interventional studies evaluated ghrelin effects on depression in humans. Contrastingly, there are demonstrations of an anti-depressant effect of ghrelin in murine models of depression induced by chronic stress^20,22,66^, what suggests that increased endogenous ghrelin levels could be a mechanism aimed at fighting the disease.

The relevance of ghrelin for depression in postmenopause also remains to be determined. The triad ghrelin/depression/estrogen relationships are probably complex, as indicated by demonstrations that blockade of estrogen receptors hindered the anti-depressant effect of ghrelin in ovariectomized mice^38^ and that, conversely, estradiol administration increased the expression of ghrelin receptors in the arcuate nucleus of female mice^67^. There is also evidence that estradiol replacement augmented ghrelin-induced stimulation of GH levels in postmenopausal women^35^.

The above data indicate that, although the majority of animal studies have indicated an anti-depressant effect of ghrelin, more studies in humans are necessary to ascertain this aspect.

It is important to clarify that the current findings may not be generalized due to the small sample size of the severe depression group. Therefore, the findings need to be interpreted cautiously pending replication in larger samples. Future researches direction should note to include a control comparison.

In summary, the present findings confirmed the existence of an association between obesity and anxiety/depression in postmenopausal women. Moreover, we demonstrated for the first time that acylated ghrelin levels associate positively with depression in this population. Further investigations are warranted to assess whether the elevated acylated ghrelin levels reflected a risk factor or a protection mechanism.

## Methods

This cross-sectional study was designed according to STROBE (Strengthening the Reporting of Observational Studies in Epidemiology) guidelines and was approved by the Ethics Committee of the Universidade Federal de São Paulo (CEP #921.394/2014). The investigation was conducted in accordance with the Declaration of Helsinki. All participants have signed the informed consent.

### Study design and study population

Two hundred and thirty-three volunteers that were recruited by the Universidade Federal de São Paulo media got in contact with our group. All volunteers were patients of the Gynecology Division of the Hospital São Paulo, the main affiliated hospital of the Universidade Federal de São Paulo. Of these, 24 refused to participate, 48 were classified with no depression symptoms, and 106 did not meet the eligibility criteria due to: premenopausal stage, age, absence of depressive symptoms, surgical menopause, hormone therapy, use of antidepressants, menopausal transition, and use of insulin. The inclusion criteria were: women aged 50–65 years, amenorrhea for at least 12 months, follicle-stimulating hormone (FSH) levels greater than 30 mUI/mL, depression symptoms, no use of hormonal replacement therapy, antidepressants or insulin. The volunteers were medically evaluated for the confirmation of the menopause status.

At the day of the study, the participants arrived between 7 and 8 a.m., after 12 h of fasting. Saliva and blood samples were collected and, then a trained dietitian performed the anthropometric assessments. The participants were then instructed about the depression and anxiety inventories and answered the questionnaires.

Fifty-five postmenopausal women with depression symptoms were included in the study and were allocated into three groups according to the classification of depression symptoms, assessed by the Beck Depression’s Inventory (BDI): mild (n = 26), moderate (n = 22) or severe depression group (n = 7). BDI is a questionnaire that evaluates the presence and severity of depression symptoms in adults. It is a self-report instrument, consisting of 21 questions with a score from 0 to 3 for each question, and with a score range of 0–63. The total score allows the classification of depressive-like symptoms as none or minimal (0–9), mild (10–16), moderate (17–29) or severe (30–63)^68^.

To reinforce the results of depression symptoms of the postmenopausal women, we also applied the Patient Health Questionnaire-9 (PHQ-9). This self-report questionnaire is composed of 9 questions about common depression symptoms, the questions being rated 0 (not at all) to 3 (nearly every day) pointing to the degree of severity of depression symptoms that the subject experienced in the previous 2 weeks. The sum of the scores indicates: no depression (0–4), mild depression (5–9), moderate depression (10–14), moderately severe depression (15–19), and severe depression (20–27)^69^.

Additionally, the Beck Anxiety's Inventory (BAI) was also applied. This questionnaire has a similar structure as BDI, containing 21 questions with four statements about anxiety-like symptoms. The classification according to BAI is as follows: minimal (0–7), mild (8–15), moderate (16–25) or severe (26–63)^70^.

### Anthropometric measurements

A trained dietitian took all the anthropometric measurements, which were assessed by a tetrapolar bioimpedance system, with 8-point tactile electrodes (InBody 230, Biospace Corp, Seoul, Korea) and 2 frequencies (20 and 100 kHz). This system has a reported intra-rater reliability of 0.12% (95% CI − 0.02, 0.27) (von Hurst et al. 2015). The data included body mass, height, BMI, body fat, percentage of body fat, SMM, FFM, WHR and BMR. The *Cunningham* equation was used to calculate BMR: 21.6 X FFM(kg) + 370 = BMR (kcal/day).

### Laboratory analysis

Samples of saliva and blood were collected after 12 h of fasting, between 7:00 and 8:00 a.m. Cotton roles (Salivette, Sarstedt AG & Co., Nümbrecht, Germany) were used to collect saliva samples that were promptly centrifuged and stored at 4 °C. Cortisol levels were determined in the same day by ELISA kit with intra-assay variation coefficient of 8.1%, inter-assay variation coefficient of 6.6%, and sensitivity of 2.5 ng/mL (Labor Diagnostika Nord, Nordhorn, Germany).

Blood was collected into dry tubes (for serum sampling after clotting) or into EDTA tubes (for plasma sampling) and centrifuged (3000×*g* for 15 min at 4 °C). Both plasma and serum were frozen at − 80 °C. Total and acylated ghrelin levels were determined in plasma, while the other hormones were determined in serum samples. For ghrelin analyses, the blood samples were collected in EDTA2Na (1 mg/mL) tubes containing 500U/mL of serine proteases inhibitor (Pefabloc, Sigma-Aldrich, St Louis, MO, USA). After centrifugation, the plasma was acidified with HCl (final concentration of 1 N). Total ghrelin (coefficient of variation (CV): intra-assay 1.1%, inter-assay 5.18%, sensitivity 50 pg/mL), active ghrelin (CV: intra-assay 0.88%, inter-assay 7.54%, sensitivity 15 pg/mL), leptin (CV: intra-assay 1.9%, inter-assay 1.3%, sensitivity 0.78–100.0 ng/mL), and adiponectin (CV: intra-assay 7.4%, inter-assay 8.4%, sensitivity 0.2 ng/mL) levels were all assayed using ELISA kits (Milliplex–Millipore, Billerica, MA). Glucose, FSH, total cholesterol, HDL, LDL, Non-LDL and triglycerides levels were determined in serum by enzymatic colorimetric methods (Labtest Diagnóstica, Lagoa Santa, MG, Brazil).

The HOMA-IR was estimated by the formula: HOMA-IR = [glucose (mmol/L) × insulin (µU/mL)/22.5]^71^.

### Statistical analysis

The SPSS software version 18.0 (IBM, Armonk, NY, USA) was used to perform all statistical analysis. The outliers were excluded according to the Chauvenet’s criterion. Data distribution was analyzed by the Shapiro–Wilk normality test, while the homogeneity of variance was assessed by Levene test. Parametric variables were analyzed by ANOVA and Tukey post hoc test. Nonparametric variables were analyzed by Kruskall–Wallis and multiple comparisons test. Relationships between hormonal/anthropometric parameters and depression/anxiety scores were determined by the Pearson’s correlation coefficient.

All the variables showing significant correlations with the psychological scores were tested in multivariate linear regression analyses to identify predictors for depression and anxiety symptoms. However, some of our data were missing at random (MAR) due to the fact that some samples were out of range for the assays used. Thus, the model for BDI included 49 women, the model for BAI included 53 women, and the model for PHQ-9 included 44 women.

The sample size of a minimum of 44 participants was calculated considering a statistical power of 0.85, an effect size of 0.31, alpha level of 0.05, and 3 predictors.

## Supplementary Information

Supplementary Table 1.
