# Supplementary material for: Preliminary evidence of acylated ghrelin association with depression severity in postmenopausal women
Source: Sci Rep. 2021 Mar 5;11:5319. doi: 10.1038/s41598-021-84431-2 (PMC7935977; doi:10.1038/s41598-021-84431-2)
Supplement: Supplementary file 1 — Supplementary Table 1. [file 41598_2021_84431_MOESM1_ESM.docx]

**Preliminary evidence of acylated ghrelin association with depression severity in postmenopausal women**

Maria Fernanda Naufel^1^, Amanda Paula Pedroso^1^, Lila Missae Oyama^1^, Mônica Marques Telles^1^, Helena Hachul^2,3^, Eliane Beraldi Ribeiro^1*^

Departments of ^1^Physiology; ^2^Psychobiology; ^3^Gynecology

Universidade Federal de São Paulo (UNIFESP), São Paulo, SP, Brazil

***Corresponding author**

Eliane Beraldi Ribeiro

Universidade Federal de São Paulo, Departamento de Fisiologia.

Rua Botucatu 862

Vila Clementino, 04023-062, São Paulo, SP, Brazil.

E-mail: [eliane.beraldi@gmail.com](mailto:eliane.beraldi@gmail.com)

Phone/Fax: 55 11 5576-4765

Supplementary table 1^#^

|  | **Correlations** | | | | | | | | | | | | |
| --- | --- | --- | --- | --- | --- | --- | --- | --- | --- | --- | --- | --- | --- |
|  | **BDI** | **BAI** | **PHQ-9** | **Total ghrelin** | **Acylated ghrelin** | **BMI** | **SMM** | **WHR** | **FFM** | **BMR** | **Insulin** | **Glucose** | **HOMA-IR** |
| **BDI** | 1.0000**^μ^**  p=---**^γ^** | 0.5311  **p<0.001** | 0.7305  **p<0.001** | 0.4842  **p<0.001** | 0.5519  **p<0.001** | 0.2558  p=0.059 | 0.1358  p=0.332 | 0.3474  **p=0.009** | 0.1364  p=0.330 | 0.1533  p=0.264 | 0.0579  p=0.680 | 0.2301  p=0.104 | 0.1629  p=0.264 |
| **BAI** | 0.5311  **p<0.001** | 1.0000  p=--- | 0.6034  **p<0.0001** | 0.2377  p=0.096 | 0.1373  p=0.347 | 0.2377  p=0.096 | 0.2909  **p=0.031** | 0.4442  **p<0.001** | 0.2970  **p=0.031** | 0.2905  **p=0.031** | 0.2460  p=0.076 | 0.2109  p=0.137 | 0.2686  p=0.062 |
| **PHQ-9** | 0.7305  **p<0.001** | 0.6034  **p<0.0001** | 1.0000  p=--- | 0.3380  **p=0.016** | 0.3400  **p=0.017** | 0.2280  p=0.093 | 0.1359  p=0.322 | 0.4142  **p=0.002** | 0.1376  p=0.326 | 0.1327  p=0.334 | 0.3715  **p=0.006** | 0.2769  **p=0.049** | 0.4315  **p=0.002** |
| **Total Ghrelin** | 0.4841  **p<0.001** | 0.2377  p=0.096 | 0.3380  **p=0.016** | 1.0000  p=--- | 0.6476  **p<0.001** | -0.0766  p=0.605 | -0.2597  p=0.075 | 0.1545  p=0.295 | -0.1266  p=0.933 | -0.1193  p=0.419 | -0.2383  p=0.139 | 0.0088  p=0.975 | -0.1869  p=248 |
| **Acylated Ghrelin** | 0.5519  **p<0.001** | 0.1373  p=0.347 | 0.3400  **p=0.017** | 0.6476  **p<0.001** | 1.0000  p=--- | -0.0712  p=0.631 | -0.1266  p=0.391 | 0.1265  p=391 | -0.2597  p=0.075 | -0.2512  p=0.085 | -0.2512  p=0.085 | 0.0516  p=0.752 | -0.1566  p=0.335 |
| **BMI** | 0.2558  **p=0.059** | 0.2377  p=0.096 | 0.2280  p=0.093 | -0.0766  p=0.605 | -0.0712  p=0.631 | 1.0000  p=--- | 0.4331  **p<0.001** | 0.5794  **p<0.001** | 0.4295  **p<0.001** | 0.4302  **p=0.001** | 0.5020  **p<0.001** | 0.2268  p=2268 | 0.4452  **p=0.002** |
| **SMM** | 0.1358  p=0.332 | 0.2909  **p=0.031** | 0.1359  p=0.322 | -0.2597  p=0.075 | -0.1266  p=0.391 | 0.4331  **p<0.001** | 1.0000  p=--- | 0.3197  **p=0.020** | 0.9991  **p<0.001** | 0.9990  **p<0.001** | 0.2795  p=0.057 | -0.1124  p=252 | 0.1704  p=0.252 |
| **WHR** | 0.3474  **p=0.009** | 0.4442  **p<0.001** | 0.4142  **p=0.002** | 0.1545  p=0.295 | 0.1265  p=391 | 0.5794  **p<0.001** | 0.3197  **p=0.020** | 1.0000  p=--- | 0.3141  **p=0.022** | 0.3148  **p=0.022** | 0.4133  **p=0.004** | 0.1319  p=0.377 | 0.3370  **p=0.021** |
| **FFM** | 0.1364  p=0.330 | 0.2970  **p=0.031** | 0.1376  p=0.326 | -0.1266  p=0.933 | -0.2597  p=0.075 | 0.4295  **p<0.001** | 0.9991  **p<0.001** | 0.3141  **p=0.022** | 1.0000  p=--- | 1.0000  **p<0.001** | 0.2624  p=0.075 | -0.1158  p=0.438 | 0.1564  p=0.294 |
| **BMR** | 0.1533  p=0.164 | 0.2905  **p=0.031** | 0.1327  p=0.334 | -0.1193  p=0.419 | -0.2512  p=0.085 | 0.4302  **p=0.001** | 0.9990  **p<0.001** | 0.3148  **p=0.022** | 1.0000  **p<0.001** | 1.0000  p=--- | 0.2621  p=0.075 | -0.1167  p=0.435 | 0.1558  p=0.296 |
| **Insulin** | 0.0579  p=0.680 | 0.2460  p=0.076 | 0.3715  **p=0.006** | -0.2383  p=0.139 | -0.2208  p=0.171 | 0.5020  **p<0.001** | 0.2795  p=0.057 | 0.4133  **p=0.004** | 0.2624  p=0.075 | 0.2621  p=0.075 | 1.0000  p=--- | 0.4666  **p=0.001** | 0.9405  **p<0.001** |
| **Glucose** | 0.2301  p=0.104 | 0.2109  p=0.137 | 0.2769  **p=0.049** | 0.0088  p=0.975 | 0.0516  p=0.752 | 0.2268  p=2268 | -0.1124  p=252 | 0.1319  p=0.377 | -0.1158  p=0.438 | -0.1167  p=0.435 | 0.4666  **p=0.001** | 1.0000  p=--- | 0.7241  **p<0.001** |
| **HOMA-IR** | 0.1629  p=0.264 | 0.2686  p=0.062 | 0.4315  **p=0.002** | -0.1869  p=248 | -0.1566  p=0.335 | 0.4452  **p=0.002** | 0.1704  p=0.252 | 0.3370  **p=0.021** | -0.1158  p=0.438 | 0.1558  p=0.296 | 0.9405  **p<0.001** | 0.7241  **p<0.001** | 1.0000  p=--- |

^#^ Only the variables showing at least one significant correlation are shown. BDI, Beck’s depression inventory; BAI, Beck’s anxiety inventory; PHQ-9, patient health questionnaire-9; BMI, body mass index; SMM, skeletal muscle mass; WHR, waist-hip ratio; FFM, fat free mass; BMR, basal metabolic rate homeostasis model assessment of insulin resistance (HOMA-IR). **^μ^**Pearson correlation; **^γ^**P-value (two-tailed). Bold values indicate statistical significance.
